# Supplementary material for: Solvent-Free Production by Extrusion of Bio-Based Poly(glycerol-co-diacids) Sheets for the Development of Biocompatible and Electroconductive Elastomer Composites
Source: Polymers (Basel). 2022 Sep 13;14(18):3829. doi: 10.3390/polym14183829 (PMC9502118; doi:10.3390/polym14183829)
Supplement: Supplementary file 1 [file polymers-14-03829-s001.zip › polymers-1846132-supplementary.pdf]

Supplementary Materials: Solvent-Free Production by Extrusion of Bio-Based Poly(Glycerol-Co-Diacid) Sheets for the Development of Biocompatible and Electroconductive Elastomer Composites

Shengzhi Ji [1], Mathilde Stricher [1,2], Frédéric Nadaud [3], Erwann Guenin [1], Christophe Egles [2,†,‡] and Frédéric Delbecq [1,*,†]

1 Université de Technologie de Compiègne, ESCOM, TIMR (Integrated Transformations of Renewable Matter), Centre de Recherche Royallieu, CS 60 319, CEDEX, 60 203 Compiègne, France
2 Université de Technologie de Compiègne, CNRS, BMBI (Biomechanics and Bioengineering), Centre de Recherche Royallieu, CS 60 319, CEDEX, 60 203 Compiègne, France
3 UTC-SAPC, Rue du Docteur Schweitzer, CS 60319, CEDEX, 60 203 Compiègne, France
* Correspondence: f.delbecq@escom.fr
† These authors contributed equally to this work.
‡ Current address: Normandie Université, UNIROUEN, INSA Rouen, CNRS, PBS (UMR 6270), 55 Rue Saint-Germain, 27 000 Évreux, France

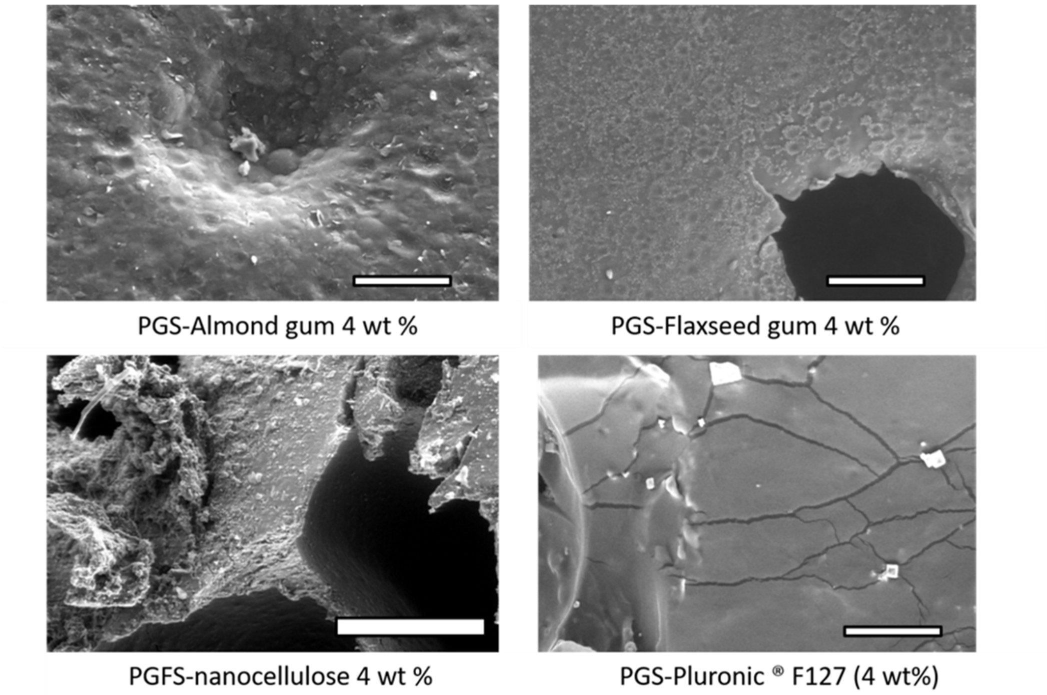

Figure S1. FE-SEM micrographs of various poly(glycerol-co-diacids) blended composites. The scale bars were set up at 400 µm.

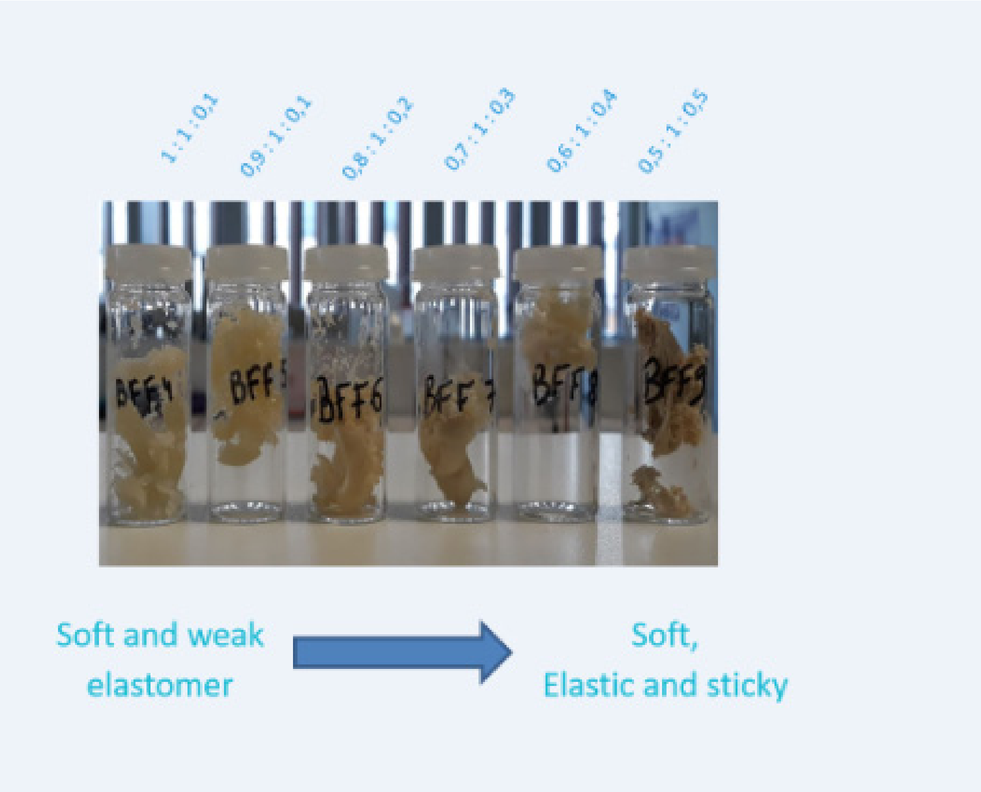

Figure S2. Evolution of PGFS aspect function of their SA and FDCA contents.

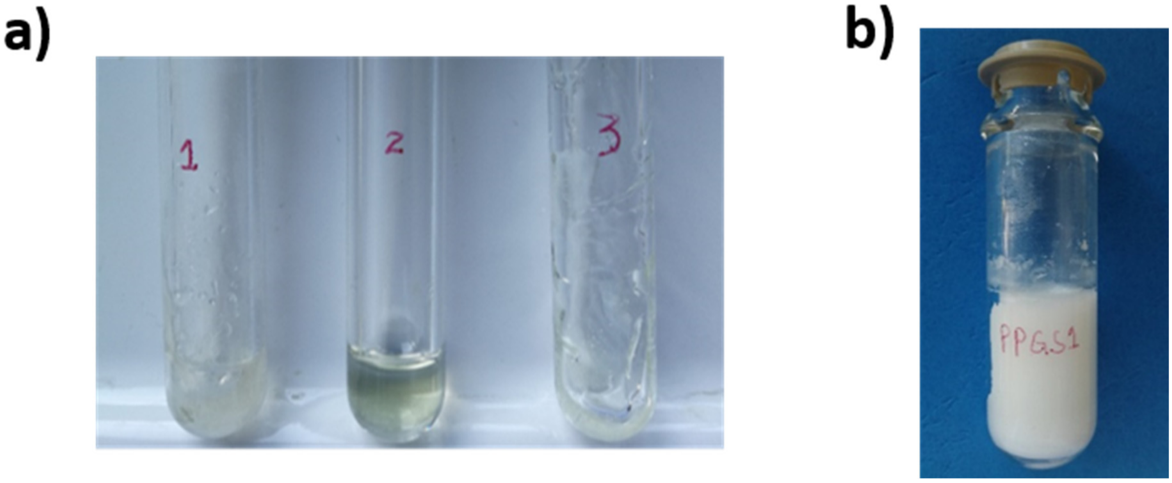

Figure S3. Pictures of: (a) PGI prepolymer formed under micro-wave heating (from left to right: 15 min without *p*TSA ; 5 min with *p*TSA ; 15 min with *p*TSA); (b) PGS prepolymer produced after 15 min of MW irradiation in presence of *p*TSA catalyst.

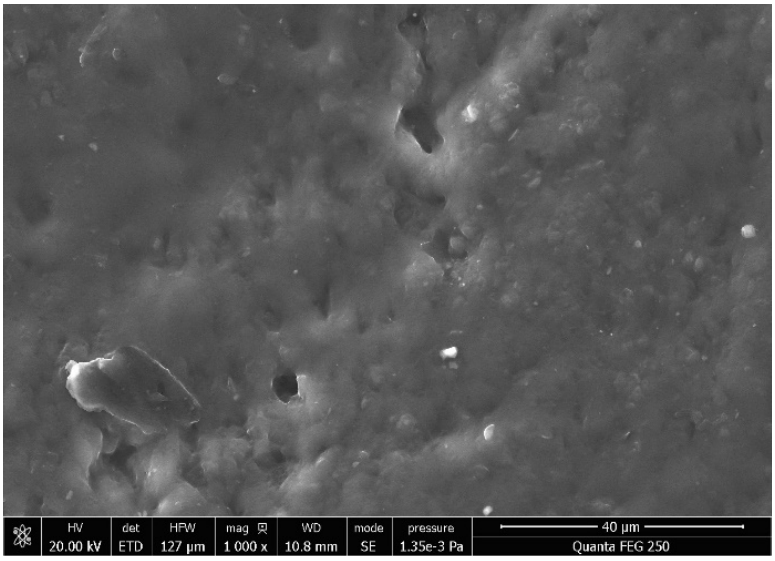

Figure S4. Picture of enlargement from PGS5 FE-SEM observation enlargement.

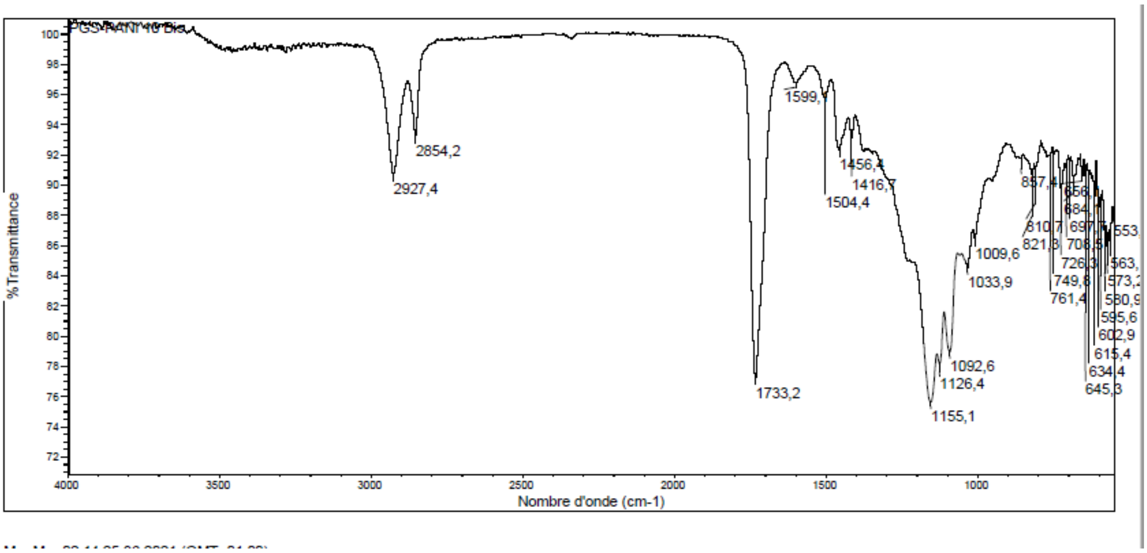

Figure S5. FT-IR spectrum of PGS9.

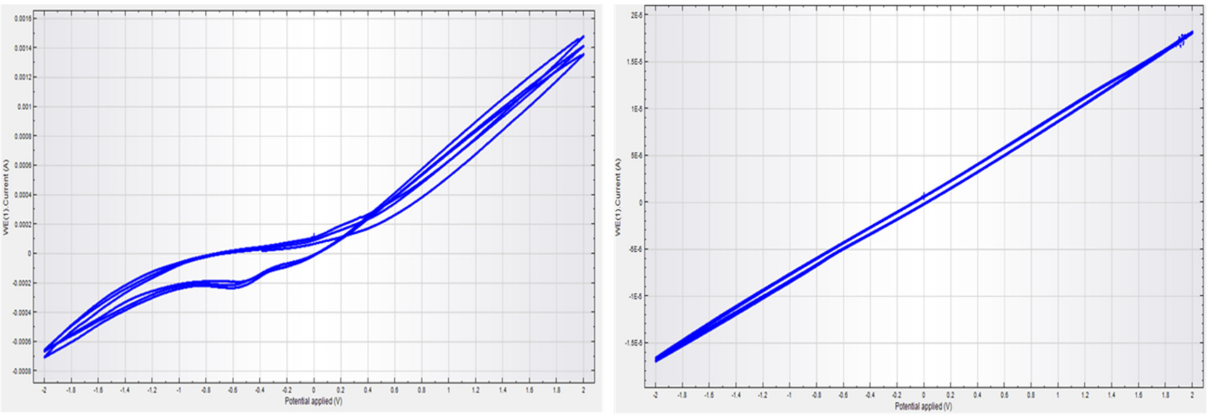

PGS8

PGS9

Figure S6. Cyclic voltammograms (CV) obtained from measurements conducted in 0.1 M $LiClO_4$ aqueous solution using PGS8 and PGS9 as working electrodes.

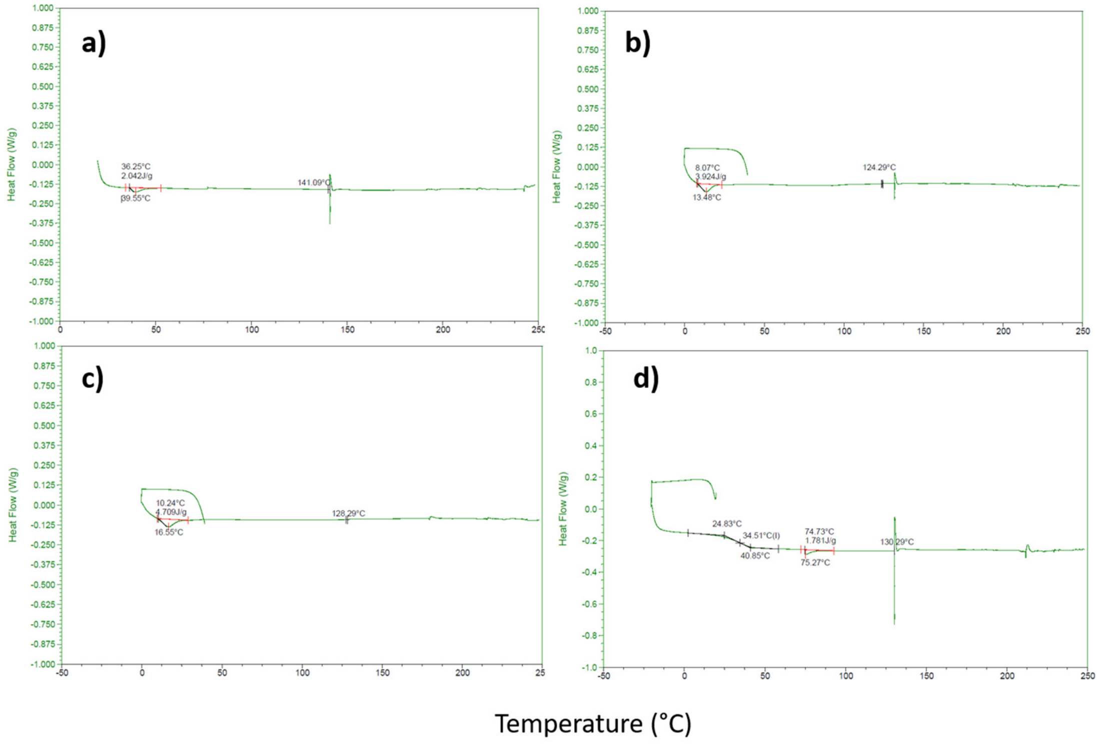

Figure S7. DSC thermograms of: (a) PGS1; (b) PGS2; (c) PGS3; (d) PGS4.

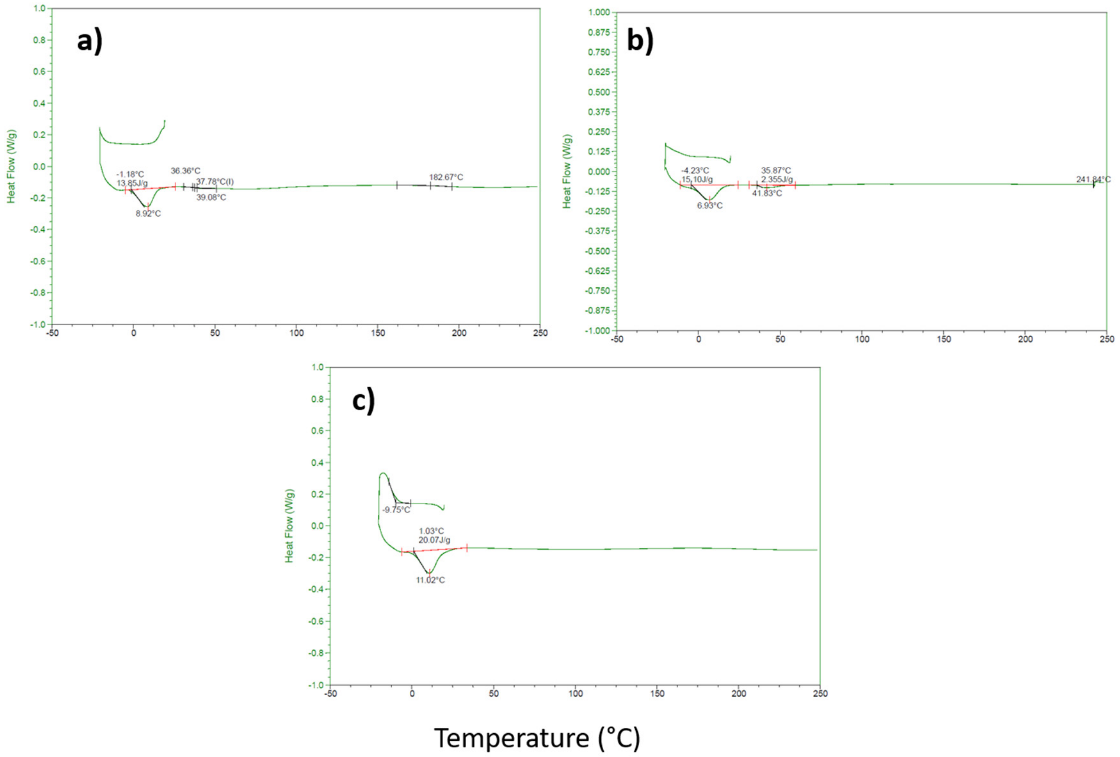

Figure S8. DSC thermograms of: (a) PGS5; (b) PGS6; (c) PGS7.

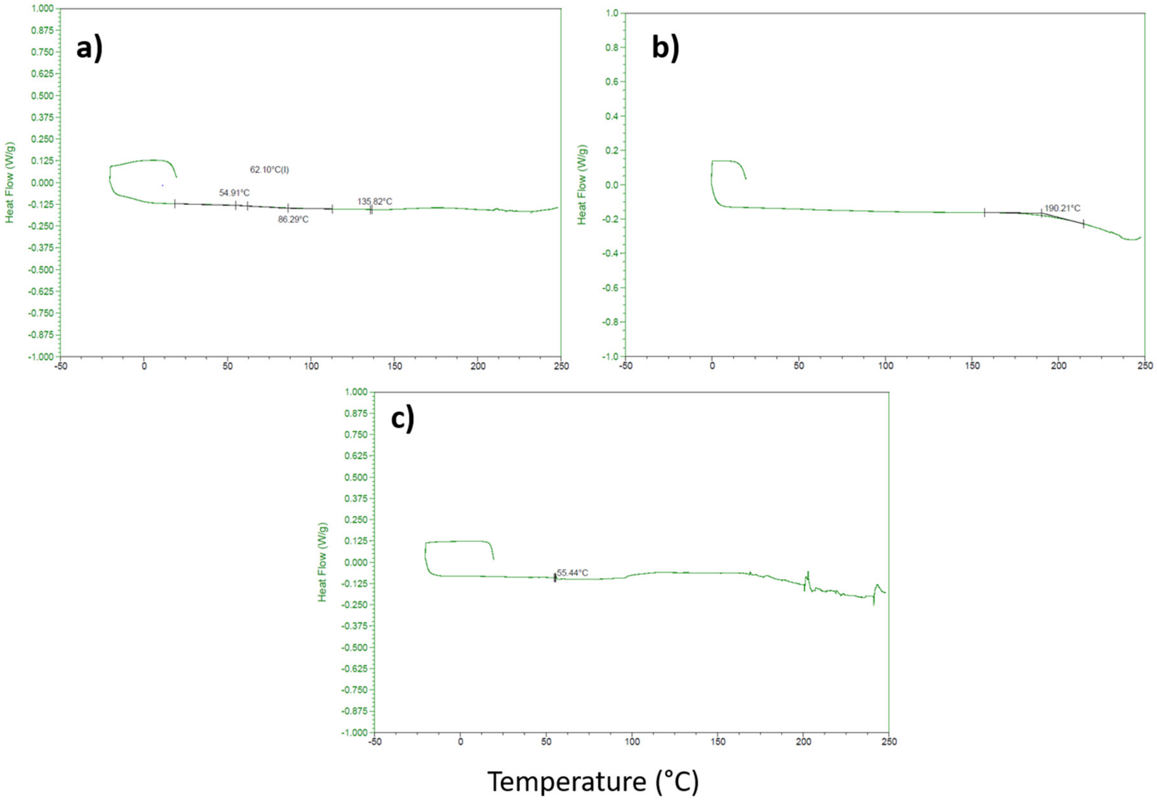

Figure S9. DSC thermograms of: (a) PGI2; (b) PGFS1; (c) PGFS2.

1. Cytotoxicity tests: General procedure for verifying cytocompatibility of polymer sheets

Extracts:

Latex (thickness < 0.5 mm) = 6 cm²/mL

1 piece of 1cm² = 2cm² (both sides counting) => 5 mL medium = 15 pieces of latex

Place 15 pieces of latex in 5 mL of medium in a 50 mL tube.

Elastomeric materials (thickness > 1mm) = 1.25 cm²/mL

PGI-Co, PGS7-Co, PGS4, PGS6, PGS7 and PGFS-Co => 5 mL = 6.25 cm²

Place material in adequate medium volume in 50 mL tube

Protocol:

- Seed L929 to 10,000 cells in 100 µL of complete medium per well of a 96-well plate. Incubate 24 hours at 37°C 5% CO_2
- Prepare the extracts using previously sterilized materials at the required concentration. Incubate 24 hours on stirring at 37°C 5% CO_2
- After 24 hours, remove the culture medium from the cells and replace with 100 µL of extract (vortexed at the end of incubation but not centrifuged). Incubate 24h at 37°C and 5% CO_2.
- After 24 h of incubation, replace the medium with an MTS mix previously heated at 37°C (CellTiter 96® AQueous One Solution Cell Proliferation Assay-PROMEGA) diluted in complete DMEM (MTS mix = 20 µL of MTS reagent + 100 µL of medium). Incubate at 37°C and 5% CO_2 for 2 hours.
- Read absorbance at 492 nm.
- Calculate cell viability: $\frac{DO_{EXTRACTS} - DO_{MEDIUM}}{DO_{CTRL-}} \times 100$
